# Longitudinal changes in each retinal layer thickness in patients with non-ischemic central retinal vein occlusion

**DOI:** 10.1186/s40662-024-00397-y

**Published:** 2024-08-01

**Authors:** Min-Woo Lee, Ji-Ho Jun, Hyun-Je Seong

**Affiliations:** 1 Department of Ophthalmology, Konyang University College of Medicine, Daejeon, Republic of Korea; 2 Department of Ophthalmology, Konyang University Hospital, #1643 Gwanjeo-Dong, Seo-Gu, Daejeon, Korea

**Keywords:** Central retinal vein occlusion, Retinal layer thickness, Optical coherence tomography

## Abstract

**Background:**

To identify longitudinal changes in each retinal layer thickness in central retinal vein occlusion (CRVO) patients with resolved macular edema (ME).

**Methods:**

In this retrospective observational study, CRVO patients without a recurrence of ME for more than 3 years and normal controls were enrolled. Each retinal layer thickness of the parafoveal area, including ganglion cell complex (GCC), inner nuclear layer (INL), outer plexiform layer (OPL), outer nuclear layer (ONL), photoreceptor layer (PRL), and retinal pigment epithelium (RPE) was measured. After the resolution of ME, three more examinations with a 1-year interval were analyzed.

**Results:**

A total of 98 eyes were enrolled, 50 eyes for the control group and 48 eyes for the CRVO group. The baseline GCC thickness was 114.2 ± 15.6 μm and 104.2 ± 25.4 μm in the control and CRVO groups, respectively, which was significantly different (*P* = 0.022). The thicknesses of other layers including INL, OPL, ONL, PRL, and RPE were not significantly different at baseline. The reduction rate of GCC, INL, OPL, and ONL was − 3.92, − 1.33, − 0.91, and − 2.31 μm/year in the CRVO group, whereas no significant reductions were observed in the control group. Best-corrected visual acuity was significantly associated with changes in the GCC, OPL, and ONL in the CRVO group.

**Conclusions:**

In patients with CRVO, even in the absence of recurrent ME, retinal damage progresses over time, evidenced by thinning of the inner retina and outer retina including OPL and ONL. These changes may be associated with alterations in visual function.

## Background

Central retinal vein occlusion (CRVO) is one of the most common retinal vascular diseases [1]. The prevalence has been reported to be 0.1% to 0.4%, and it is known to be more common in individuals with arteriosclerosis, diabetes, and hypertension [2–4]. The upregulation of vascular endothelial growth factor (VEGF) expression in the retinal non-perfusion area increases vascular permeability, leading to macular edema (ME) and subsequent vision loss [5]. Since the development of anti-VEGF treatments, various clinical trials have demonstrated their efficacy in CRVO accompanied by ME [6, 7]. Intravitreal steroid injections are also considered effective treatments for ME in CRVO patients [8].

Although anti-VEGF and steroid treatments may lead to the improvement of ME, CRVO-induced retinal damage may not fully recover. Podkowinski et al. [9] reported neuroretinal atrophy in CRVO eyes with resolved ME after ranibizumab therapy. Kim et al. [10] demonstrated a reduction of inner retinal layer thickness in ME eyes compared with non-ME eyes; the minimum ganglion cell-inner plexiform layer (GC-IPL) thickness was correlated with the visual acuity in non-ischemic CRVO. Their findings suggested that inner retinal damage can result in permanent visual impairment after treatment. Therefore, CRVO-induced retinal thinning, potentially associated with visual function, may manifest as retinal damage despite the absence of ME. However, to our knowledge, there is a lack of longitudinal studies regarding each retinal layer thickness in CRVO patients with resolved ME.

The purpose of this study was to explore longitudinal changes in the thickness of each retinal layer in CRVO patients with resolved ME.

## Methods

### Patients

This retrospective, longitudinal, observational study adhered to the tenets of the Declaration of Helsinki; the study protocol was approved by the Institutional Review Board/Ethics Committee of Konyang University Hospital, Daejeon, Republic of Korea (No. 2024–03-008). Patients who attended our retinal clinic between March 2017 and December 2023 were screened for inclusion. The requirement for informed consent was waived by the Institutional Review Board/Ethics Committee of Konyang University Hospital due to the retrospective nature of the study. Patients with CRVO who had not experienced ME recurrence for ≥ 3 years after anti-VEGF treatment were included. To analyze the association between visual acuity and retinal thickness changes, patients with ischemic CRVO (presence of ≥ 10 disc areas of retinal capillary nonperfusion on fundus fluorescein angiography) who typically experienced persistent severe visual impairment were excluded [11]. The control group included patients who had been diagnosed with unilateral epiretinal membrane, macular hole, or intraocular lens dislocation. Fellow eyes without any ophthalmic pathology were included for comparative analysis.

After ME resolution, three additional examinations at 1-year intervals were performed and analyzed. The exclusion criteria were a history of ocular surgery except cataract extraction, ocular diseases other than CRVO, intraocular pressure (IOP) > 21 mmHg, axial length ≥ 26 mm, and optic disc pathology. Patients who previously received or required photocoagulation treatment during the study period were also excluded.

### Optical coherence tomography (OCT)

**Fig. 1:**
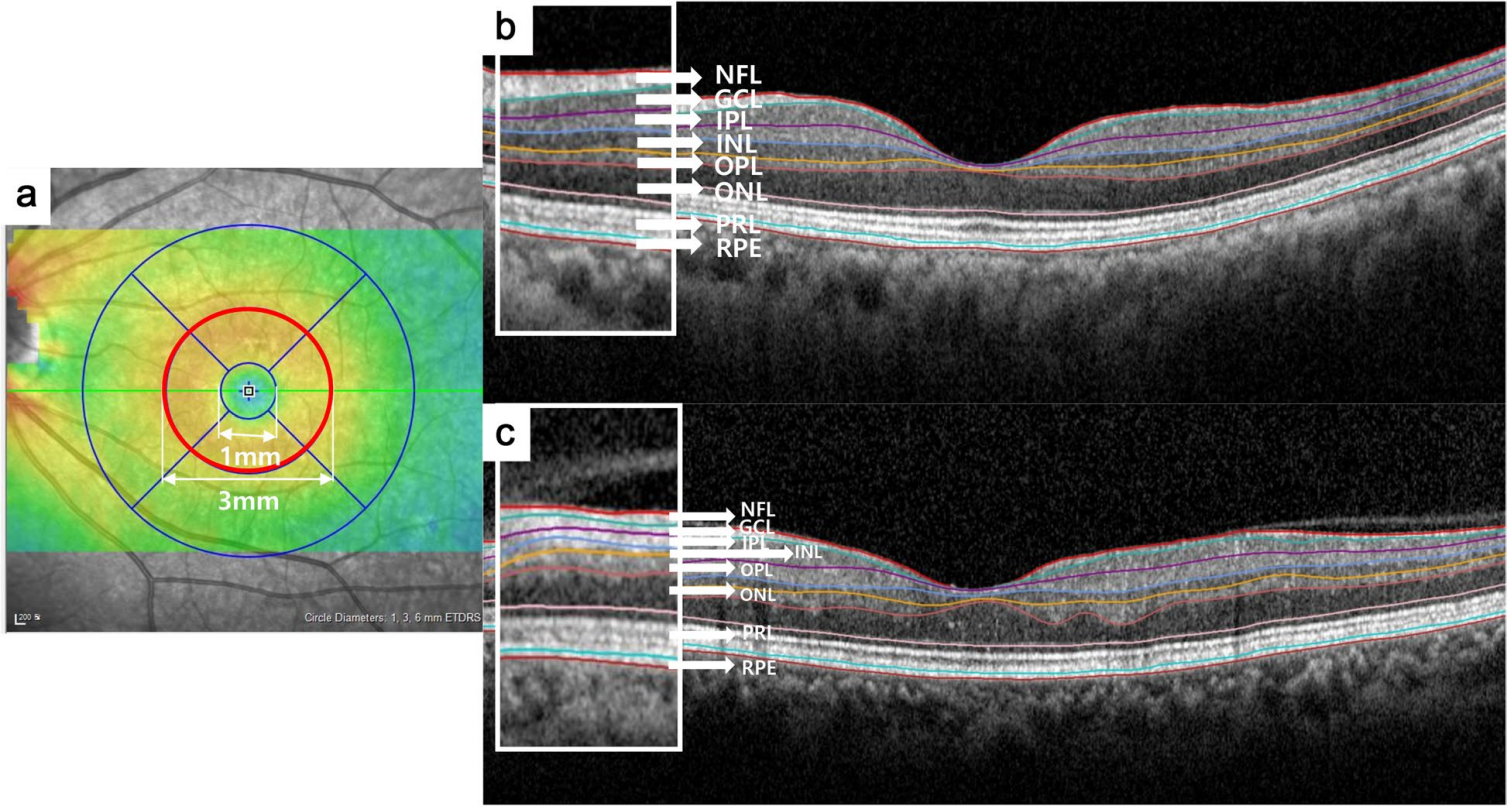
Macular regions analyzed by spectral-domain optical coherence tomography scan in the retinal thickness map analysis. **a** The thickness of the parafoveal area (red circle) ranging from 1 to 3 mm from the subfoveal region was analyzed. Representative B-scan images of the control group (**b**) and the CRVO group (**c**). The boundaries of the fundus structure were segmented by an automated algorithm. CRVO, central retinal vein occlusion; NFL, nerve fiber layer; GCL, ganglion cell layer; IPL, inner plexiform layer; INL, inner nuclear layer; OPL, outer plexiform layer; ONL, outer nuclear layer; PRL, photoreceptor layer; RPE, retinal pigment epithelium

OCT measurements were performed by a skilled examiner with spectral-domain OCT (SD-OCT; Spectralis; Heidelberg Engineering, Heidelberg, Germany), using a volume scan of 25 horizontal line scans (512 A-scans per B-scan, 245 μm interscan distance) with automatic real-time mode averaging six images. Retinal thickness map analyses were used to establish numeric averages of the measurements for the nine Early Treatment Diabetic Retinopathy Study (ETDRS) subfields in assessments of retinal layer thickness. To examine changes in each retinal layer thickness over time in detail, we analyzed the parafoveal area (intermediate ring of ETDRS subfields, ranging from 1 to 3 mm from the subfoveal region), which is characterized by a relatively thicker inner retinal layer. Automated retinal layer segmentation was performed using the built-in software, Heidelberg Eye Explorer ver. 6.9a (Heidelberg Engineering, Heidelberg, Germany). The thicknesses of the ganglion cell complex [GCC, including retinal nerve fiber layer, ganglion cell layer, and inner plexiform layer (IPL)], inner nuclear layer (INL), outer plexiform layer (OPL), outer nuclear layer (ONL), photoreceptor layer (PRL), and retinal pigment epithelium (RPE) were measured (Fig. 1). Two independent investigators (M.W.L. and J.H.J.) checked OCT images, and manual adjustment was performed when an obvious segmentation error was found. Images with a quality score below 15 were excluded; images with decentration, misalignment, or severe segmentation errors were also excluded.

### Statistical analysis

Baseline demographic characteristics and ocular parameters were compared using independent t-tests. For categorical variables, the Chi-squared test was employed to compare two groups. To compare each retinal layer thickness with the previous measurements in the CRVO group, paired t-tests were used. *P* values were adjusted using Bonferroni correction. Linear mixed models were used to identify significant changes in each retinal layer over time for each group, along with the reduction rate of each retinal layer thickness. Each retinal layer thickness was fitted with linear mixed models with best-corrected visual acuity (BCVA) and follow-up duration as fixed effects. A random intercept was included at the eye level. In the CRVO group, generalized linear mixed models were used to identify factors associated with changes in each retinal layer thickness over time. Statistical analyses were performed using the SPSS Statistics software (version 18.0; IBM Corp., Armonk, NY, USA).

## Results

### Demographics

In total, 98 eyes were included – 50 in the control group and 48 in the CRVO group. BCVA values were 0.05 ± 1.0 logMAR and 0.46 ± 0.57 logMAR in the control and CRVO groups, respectively (*P* < 0.001; Table 1). The remaining baseline characteristics, including age, sex, diabetes, hypertension, spherical equivalent, IOP, and axial length, did not significantly differ between the two groups. All data were confirmed to follow a normal distribution using the Shapiro–Wilk test. In the CRVO group, the duration from CRVO onset to baseline was 12.3 ± 7.8 months. The mean interval between the final intravitreal injection and baseline was 5.6 ± 10.7 months, and the mean number of previous intravitreal injections was 3.7 ± 2.9.

**Table 1:** Baseline demographic characteristics

Parameter	Control group (*n* = 50)	CRVO group (*n* = 48)	*P* value
Age (year)	67.4 ± 13.5	68.9 ± 15.2	0.230
Sex (male, %)	34 (68.0)	25 (52.1)	0.108
Laterality (right, %)	24 (48.0)	26 (54.2)	0.542
Diabetes (n, %)	12 (24.0)	8 (16.7)	0.368
Hypertension (n, %)	8 (16.0)	14 (29.2)	0.060
Lens status (pseudophakic, %)	8 (16.0)	7 (14.6)	0.846
Spherical equivalent (diopter)	− 0.60 ± 2.36	− 0.44 ± 1.77	0.715
Intraocular pressure (mmHg)	13.9 ± 3.4	14.4 ± 4.0	0.492
Axial length (mm)	24.0 ± 1.4	24.1 ± 1.3	0.862
BCVA (logMAR)	0.05 ± 0.10	0.46 ± 0.57	**< 0.001**
CMT (μm)	268.7 ± 24.2	265.8 ± 32.2	0.608

Data are shown as the mean ± SD unless otherwise indicated

Values in boldface (*P* < 0.050) are statistically significant

*CRVO* = central retinal vein occlusion; *BCVA* = best-corrected visual acuity; *CMT* = central macular thickness

### Each retinal layer thickness at each visit

**Fig. 2:**
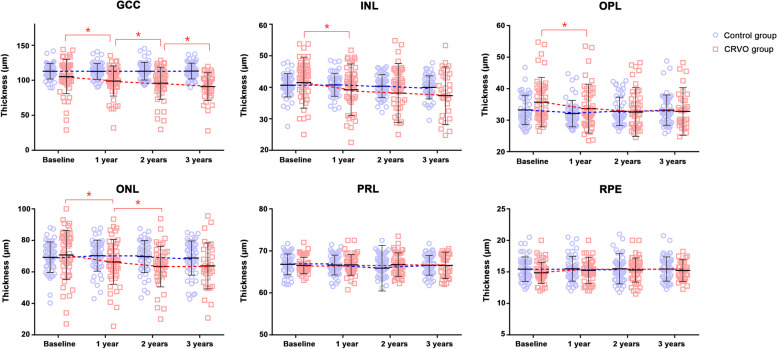
Scatter plots and line graphs showing the means and standard deviations of each retinal layer thickness at each visit. The thickness of ganglion cell complex (GCC) (*P* < 0.001), inner nuclear layer (INL) (*P* < 0.001), outer plexiform layer (OPL) (*P* < 0.001), and outer nuclear layer (ONL) (*P* < 0.001) significantly decreased over time in the central retinal vein occlusion (CRVO) group but not in the control group. The photoreceptor layer (PRL) and retinal pigment epithelium (RPE) thicknesses did not significantly change in either group. * Indicates statistically significant differences

In the control group, the quality score of OCT images was 33.1 ± 2.1, 33.2 ± 2.0, 32.9 ± 2.3, and 33.0 ± 2.2 dB and the central macular thickness was 268.8 ± 24.2, 271.5 ± 22.7, 269.3 ± 23.4, and 269.3 ± 23.4 μm at baseline, 1 year, 2 years, and 3 years, respectively (*P* = 0.642). In the CRVO group, the quality score was 32.8 ± 2.0, 33.2 ± 1.9, 32.9 ± 2.1, and 33.1 ± 2.3 dB and the central macular thickness was 265.8 ± 32.2, 263.1 ± 35.7, 260.4 ± 36.7, and 250.1 ± 34.3 μm, respectively (*P* = 0.005). Thus, there was a significant reduction over time only in the CRVO group. Baseline GCC thicknesses were 114.2 ± 15.6 and 104.2 ± 25.4 μm in the control and CRVO groups, respectively (*P* = 0.022; Table 2). The thicknesses of the remaining layers, including INL, OPL, ONL, PRL, and RPE, did not significantly differ at baseline. The GCC, INL, OPL, and ONL thicknesses significantly decreased over time in the CRVO group, but not in the control group. The PRL and RPE thicknesses did not significantly change over time in either group (Fig. 2).

**Table 2:** Parafoveal thickness in each retinal layer at each visit

Parameter	Control group	CRVO group	*P* value^*^
GCC (μm)			
Baseline	114.2 ± 15.6	104.2 ± 25.4	**0.022**
First year	112.7 ± 15.6	98.3 ± 22.1	
Second year	112.9 ± 12.6	95.8 ± 24.2	
Third year	113.1 ± 11.2	90.6 ± 20.8	
*P* value^†^	0.695	**< 0.001**	
INL (μm)			
Baseline	40.7 ± 3.7	41.6 ± 8.3	0.466
First year	40.7 ± 3.6	39.4 ± 8.4	
Second year	40.4 ± 3.6	38.3 ± 9.5	
Third year	40.0 ± 3.7	37.2 ± 9.1	
*P* value^†^	0.084	**< 0.001**	
OPL (μm)			
Baseline	33.3 ± 4.7	35.5 ± 7.8	0.080
First year	32.1 ± 4.2	33.6 ± 7.7	
Second year	32.8 ± 4.5	32.5 ± 7.7	
Third year	33.2 ± 4.8	32.3 ± 7.1	
*P* value^†^	0.783	**< 0.001**	
ONL (μm)			
Baseline	69.3 ± 9.7	69.9 ± 15.9	0.817
First year	70.3 ± 9.8	65.6 ± 13.9	
Second year	69.7 ± 10.2	62.6 ± 12.5	
Third year	68.8 ± 10.8	62.0 ± 14.2	
*P* value^†^	0.295	**< 0.001**	
PRL (μm)			
Baseline	66.8 ± 2.5	66.5 ± 1.9	0.566
First year	66.6 ± 2.4	66.7 ± 2.5	
Second year	65.9 ± 5.4	66.7 ± 2.8	
Third year	66.6 ± 2.3	66.6 ± 3.1	
*P* value^†^	0.318	0.863	
RPE (μm)			
Baseline	15.4 ± 1.9	15.3 ± 4.2	0.814
First year	15.5 ± 2.0	15.5 ± 3.5	
Second year	15.5 ± 2.4	16.3 ± 5.5	
Third year	15.5 ± 1.9	16.1 ± 6.3	
*P* value^†^	0.991	0.092	

Values in boldface (*P* < 0.050) are statistically significant

*CRVO* = central retinal vein occlusion; *GCC* = ganglion cell complex; *INL* = inner nuclear layer; *OPL* = outer plexiform layer; *ONL* = outer nuclear layer; *PRL* = photoreceptor layer; *RPE* = retinal pigment epithelium

^*^Independent t-test for baseline values

^†^Linear mixed model

### Reduction rate and associated factors for each retinal layer reduction in the CRVO group

The reduction rates for GCC, INL, OPL, and ONL were − 3.92, − 1.33, − 0.91, and − 2.31 μm/year, respectively, in the CRVO group; these rates significantly differed from rates in the control group (Table 3). The reduction rates for PRL and RPE were − 0.05 and 0.67 μm/year, respectively, in the CRVO group; these rates were not statistically significant. Age (B = − 0.63, *P* = 0.003) and BCVA (B = − 7.78, *P* = 0.009) were significantly associated with GCC reduction in the CRVO group (Table 4). Additionally, BCVA was significantly associated with reductions in OPL (B = − 1.81, *P* = 0.047) and ONL (B = − 7.68, *P* < 0.001). None of the investigated factors were significantly associated with the changes in PRL and RPE thicknesses.

**Table 3:** Rates of change in each retinal layer thickness, calculated using linear mixed models

Parameter	Control group	CRVO group	^*^P value
GCC	0.13 (− 0.54 to 0.80)	− 3.92 (− 5.53 to − 2.30)	**< 0.001**
INL	− 0.22 (− 0.47 to 0.03)	− 1.33 (− 1.83 to − 0.83)	**0.001**
OPL	0.05 (− 0.29 to 0.39)	− 0.91 (− 1.36 to − 0.46)	**0.021**
ONL	− 0.20 (− 0.57 to 0.17)	− 2.31 (− 3.45 to − 1.17)	**< 0.001**
PRL	− 0.08 (− 0.24 to 0.08)	− 0.05 (− 0.34 to 0.23)	0.939
RPE	− 0.01 (− 0.10 to 0.10)	0.67 (− 0.12 to 1.46)	0.159

Values in boldface (*P* < 0.050) are statistically significant

*CRVO* = central retinal vein occlusion; *GCC* = ganglion cell complex; *INL* = inner nuclear layer; *OPL* = outer plexiform layer; *ONL* = outer nuclear layer; *PRL* = photoreceptor layer; *RPE* = retinal pigment epithelium

^*^Interaction between group and duration in linear mixed models

**Table 4:** Linear mixed-effect model determination of factors associated with retinal layer thickness changes, showing significant reduction over time in the CRVO group

Parameter	GCC		INL		OPL		ONL	
	B (95% CI)	*P* value	B (95% CI)	*P* value	B (95% CI)	*P* value	B (95% CI)	*P* value
Age	− 0.63 (− 1.02, − 0.23)	**0.003**	− 0.07 (− 0.23, 0.08)	0.359	− 0.11 (− 0.25, 0.03)	0.111	0.10 (− 0.13, 0.34)	0.387
Sex	9.65 (− 3.57, 22.88)	0.148	− 0.58 (− 5.27, 4.11)	0.804	3.12 (− 0.99, 7.23)	0.133	3.30 (− 3.77, 10.36)	0.353
DM	10.13 (− 6.99, 27.56)	0.239	2.62 (− 3.62, 8.86)	0.403	0.44 (− 5.21, 6.09)	0.876	0.51 (− 9.09, 10.11)	0.915
HTN	− 0.62 (− 14.62, 13.38)	0.929	2.95 (− 1.94, 7.85)	0.231	1.15 (− 3.30, 5.59)	0.606	2.94 (− 4.54, 10.43)	0.433
In.No	− 0.30 (− 3.35, 2.75)	0.843	0.73 (− 0.09, 1.57)	0.082	− 0.02 (− 0.77, 0.73)	0.952	− 0.62 (− 1.88, 0.64)	0.327
Duration	− 0.08 (− 0.53, 0.36)	0.703	0.055 (− 0.11, 0.21)	0.502	0.01 (− 0.14, 0.15)	0.931	0.03 (− 0.21, 0.27)	0.800
SE	0.84 (− 3.18, 4.87)	0.675	− 1.04 (− 2.38, 0.29)	0.123	− 0.73 (− 1.94, 0.48)	0.232	− 0.57 (− 2.66, 1.51)	0.583
IOP	− 0.24 (− 1.92, 1.43)	0.773	0.48 (− 0.09, 1.05)	0.094	0.12 (− 0.41, 0.64)	0.659	− 0.48 (− 1.36, 0.40)	0.275
AXL	7.84 (− 24.11, 39.80)	0.492	− 0.56 (− 4.95, 3.85)	0.748	0.45 (− 5.70, 6.61)	0.857	− 6.88 (− 18.66, 4.90)	0.193
BCVA	− 7.78 (− 13.62, − 1.97)	**0.009**	2.07 (− 0.03, 4.16)	0.054	− 1.81 (− 3.59, − 0.02)	**0.047**	− 7.68 (− 11.13, − 4.23)	**< 0.001**

Values in boldface (*P* < 0.050) are statistically significant

*CRVO* = central retinal vein occlusion; *GCC* = ganglion cell complex; *INL* = inner nuclear layer; *OPL* = outer plexiform layer; *ONL* = outer nuclear layer; *DM* = diabetes mellitus; *HTN* = hypertension; *In.No.* = number of previous injections; *Duration* = duration from CRVO onset to baseline; *SE* = spherical equivalent; *IOP* = intraocular pressure; *AXL* = axial length; *BCVA* = best-corrected visual acuity

## Discussion

Retinal thinning is frequently observed in patients who exhibited persistent CRVO without recurrent ME. Previous studies also revealed inner retinal layer thinning among CRVO patients [9, 10]. However, longitudinal analyses of changes in the inner retinal layer have rarely been reported, and no analyses of outer retinal layer thickness have been reported thus far. The present study investigated longitudinal changes in each retinal layer thickness in patients with CRVO without recurrent ME over 3 years, revealing significant reductions over time in the GCC, INL, OPL, and ONL. Additionally, changes in the GCC, OPL, and ONL were significantly associated with BCVA.

At baseline, GCC thickness was significantly thinner in the CRVO group than in the control group. Because of its high vascularity, the GCC is expected to rapidly respond to anti-VEGF treatment, exhibiting drastic changes in thickness. The rich vasculature of this layer is presumed to play an important role in its formation and structural maintenance; CRVO-induced hypoperfusion may contribute to its thinning. Additionally, its high oxygen demand increases vulnerability to hypoxic damage, leading to early retinal thinning. In contrast, the INL, OPL, and INL in the CRVO group tended to display increased thickness compared with the control group, although these findings were not statistically significant. Because these layers exhibit less vascularity or avascularity, they may require longer intervals to recover baseline thickness compared to the GCC. The PRL and RPE, which receive a portion of their oxygen supply from the choroid, did not exhibit significant differences between the two groups or changes over time.

The GCC, thinner in the CRVO group than in the control group from a relatively early stage after ME resolution, continued to display significant thinning over 3 years. Previous longitudinal studies have demonstrated sustained inner retinal damage due to vascular diseases, such as diabetes or hypertension [12, 13]. However, continued inner retinal damage in CRVO has rarely been reported. CRVO-affected retinas appear to experience continuous inner retinal damage, despite the absence of ME recurrence. Therefore, these changes should be considered when analyzing inner retinal layer thickness in CRVO patients. Notably, Roh et al. [14] reported that parafoveal inner retinal thinning after ME resolution by anti-VEGF treatment was predictive of a lower risk of ME recurrence in CRVO. Since we included patients who did not experience ME recurrence for an extended interval, many patients with advanced inner retinal thinning may have been present among the CRVO patients in this study.

A previous study showed that 12.6% of CRVO patients exhibited retinal atrophy, predominantly in the IPL to ONL, 6 months after ME resolution [9]. Although the study revealed thinning of the IPL, INL, OPL, and ONL in a small subset of patients, the examination period was short. In contrast, the present study demonstrated significant decreases in INL, OPL, and ONL thicknesses over 3 years. These decreases may be involved in the process of ME recovery but could also result from retinal damage because thickness changes were negatively associated with BCVA. Thinning of these layers may be associated with severe impairment of the deep retinal capillary plexus in CRVO eyes due to hypoxic damage caused by impaired deep retinal capillary plexus [15]. These layers exhibited slower and less pronounced changes compared with the GCC. Notably, the rate of thickness reduction in these layers decreased each year. Unlike the continuously decreasing GCC thickness, these layers appeared to partially plateau after thinning. Therefore, although the INL, OPL, and ONL were also affected by CRVO, the GCC is likely to experience the greatest long-term impact.

Previous studies have demonstrated significant associations between inner retinal layer thickness and visual acuity in CRVO patients. Cicinelli et al. [16] found that reduced inner retinal thickness was correlated with worse visual acuity after ME resolution in RVO patients. Similarly, Zheng et al. [17] identified a significant association between mean GC-IPL thickness and visual acuity in RVO eyes with resolved ME. Consistent with these findings, our study demonstrated that changes in the GCC were significantly associated with BCVA. Changes in the OPL and ONL also were significantly associated with BCVA; these relationships have not been previously reported. These results indicate that the continuous thinning of each retinal layer due to sustained damage by CRVO, despite ME resolution, may adversely affect visual function. Because the OPL and ONL tend to stabilize after 2 years, continued thinning in the GCC may have more pronounced long-term impacts on visual function. Further longitudinal studies with over extended intervals are required to confirm this hypothesis.

This study had several limitations. First, its retrospective nature inevitably led to selection bias. Secondly, the absence of assessments (e.g., visual field tests, color vision evaluations, and contrast sensitivity analyses) limited our ability to comprehensively evaluate changes in visual function associated with altered retinal layer thicknesses. Third, due to a substantial amount of missing data, we were unable to conduct a comparative analysis with the contralateral eyes of the CRVO group. Fourth, we were unable to assess the microvasculature status of each retinal capillary plexus using OCT angiography, which could be associated with changes in retinal thickness. Nevertheless, the strength of the present study lies in its longitudinal analysis, which revealed sustained damage to each retinal layer over 3 years in patients with resolved ME after CRVO; this phenomenon has not previously been reported.

## Conclusions

In conclusion, even after ME resolution in CRVO patients, the GCC, INL, OPL, and ONL continued to thin over a 3-year period, indicating persistent retinal damage in the absence of ME recurrence. Furthermore, changes in the thicknesses of the GCC, OPL, and ONL were significantly associated with visual acuity. Although the OPL and ONL tended to stabilize by the 3-year follow-up, the GCC exhibited continued thinning, suggesting that changes in the GCC have the greatest long-term effects on visual acuity. These findings should be considered when assessing retinal thickness changes in CRVO patients.

## Data Availability

The datasets used and/or analyzed during the current study available from the corresponding author upon reasonable request.

## References

[1] Ip M, Hendrick A. Retinal vein occlusion review. Asia Pac J Ophthalmol (Phila). 2018;7(1):40–5. 29280368 10.22608/APO.2017442

[2] Song P, Xu Y, Zha M, Zhang Y, Rudan I. Global epidemiology of retinal vein occlusion: a systematic review and meta-analysis of prevalence, incidence, and risk factors. J Glob Health. 2019;9(1):010427. 31131101 10.7189/jogh.09.010427 PMC6513508

[3] McIntosh RL, Rogers SL, Lim L, Cheung N, Wang JJ, Mitchell P, et al. Natural history of central retinal vein occlusion: an evidence-based systematic review. Ophthalmology. 2010;117(6):1113–23.e15. 20430446 10.1016/j.ophtha.2010.01.060

[4] Rogers S, McIntosh RL, Cheung N, Lim L, Wang JJ, Mitchell P, et al. The prevalence of retinal vein occlusion: pooled data from population studies from the United States, Europe, Asia, and Australia. Ophthalmology. 2010;117(2):313–9.e1. 20022117 10.1016/j.ophtha.2009.07.017 PMC2945292

[5] Campochiaro PA, Hafiz G, Shah SM, Nguyen QD, Ying H, Do DV, et al. Ranibizumab for macular edema due to retinal vein occlusions: implication of VEGF as a critical stimulator. Mol Ther. 2008;16(4):791–9. 18362932 10.1038/mt.2008.10

[6] Babiuch AS, Han M, Conti FF, Wai K, Silva FQ, Singh RP, et al. Association of disorganization of retinal inner layers with visual acuity response to anti-vascular endothelial growth factor therapy for macular edema secondary to retinal vein occlusion. JAMA Ophthalmol. 2019;137(1):38–46. 30286219 10.1001/jamaophthalmol.2018.4484 PMC6440246

[7] Thach AB, Yau L, Hoang C, Tuomi L. Time to clinically significant visual acuity gains after ranibizumab treatment for retinal vein occlusion: BRAVO and CRUISE trials. Ophthalmology. 2014;121(5):1059–66. 24424249 10.1016/j.ophtha.2013.11.022

[8] Bashshur ZF, Ma’luf RN, Allam S, Jurdi FA, Haddad RS, Noureddin BN. Intravitreal triamcinolone for the management of macular edema dueto nonischemic central retinal vein occlusion. Arch Ophthalmol. 2004;122(8):1137–40. 15302653 10.1001/archopht.122.8.1137

[9] Podkowinski D, Philip AM, Vogl WD, Gamper J, Bogunovic H, Gerendas BS, et al. Neuroretinal atrophy following resolution of macular oedema in retinal vein occlusion. Br J Ophthalmol. 2019;103(1):36–42. 29511062 10.1136/bjophthalmol-2017-311614

[10] Kim HJ, Yoon HG, Kim ST. Correlation between macular ganglion cell-inner plexiform layer thickness and visual acuity after resolution of the macular edema secondary to central retinal vein occlusion. Int J Ophthalmol. 2018;11(2):256–61. 29487816 10.18240/ijo.2018.02.13 PMC5824081

[11] Group CVOS. A randomized clinical trial of early panretinal photocoagulation for ischemic central vein occlusion: the Central Vein Occlusion Study Group N Report. Ophthalmology. 1995;102(10):1434–44. 9097789 10.1016/S0161-6420(95)30848-2

[12] Lee WH, Lee MW, Lim HB, Kim KM, Shin YI, Kim JY, et al. Longitudinal changes in the thickness of the ganglion cell-inner plexiform layer in patients with hypertension: a 4-year prospective observational study. Acta Ophthalmol. 2020;98(4):e479–86. 31658412 10.1111/aos.14291

[13] Lim HB, Shin YI, Lee MW, Koo H, Lee WH, Kim JY. Ganglion cell-inner plexiform layer damage in diabetic patients: 3-year prospective, longitudinal, observational study. Sci Rep. 2020;10(1):1470. 32001760 10.1038/s41598-020-58465-x PMC6992712

[14] Roh HC, Lee GW, Kang SW, Son KY, Kang MC, Choi KJ, et al. Parafoveal inner retinal thinning as the biomarker predicting less recurrence of macular edema in central retinal vein occlusion after discontinuing antivascular endothelial growth factor. Retina. 2022;42(12):2336–45. 36394888 10.1097/IAE.0000000000003616

[15] Coscas F, Glacet-Bernard A, Miere A, Caillaux V, Uzzan J, Lupidi M, et al. Optical coherence tomography angiography in retinal vein occlusion: evaluation of superficial and deep capillary plexa. Am J Ophthalmol. 2016;161:160–71.e1–2. 26476211 10.1016/j.ajo.2015.10.008

[16] Cicinelli MV, La Franca L, Berni A, Bottazzi L, Rabiolo A, Lattanzio R, et al. Rate and associations of inner retinal thinning in eyes with retinal vein occlusion and regressed macular oedema. Eye (Lond). 2024;38(1):138–44. 37391514 10.1038/s41433-023-02647-0 PMC10764826

[17] Zheng Z, Yan M, Li L, Zhang D, Zhang L. Analysis of ganglion cell-inner plexiform layer thickness in retinal vein occlusion with resolved macular edema. Int Ophthalmol. 2023;43(2):655–64. 36411372 10.1007/s10792-022-02569-y

